# Angiographic characteristics of vasculopathy in patients with idiopathic inflammatory myopathies and systemic sclerosis

**DOI:** 10.1038/s41598-022-12991-y

**Published:** 2022-05-31

**Authors:** Jina Yeo, Eun-Ah Park, Eun Bong Lee, Jin Kyun Park

**Affiliations:** 1 Division of Rheumatology, Department of Internal Medicine, Gil Medical Center, Gachon University College of Medicine, Incheon, Republic of Korea; 2 Division of Rheumatology, Department of Internal Medicine, Seoul National University Hospital and College of Medicine, 101 Daehak-ro, Jongno-gu, Seoul, 03080 Republic of Korea; 3 Department of Radiology, Seoul National University Hospital and College of Medicine, Seoul, Republic of Korea

**Keywords:** Radiography, Connective tissue diseases, Vasculitis syndromes

## Abstract

To describe the peripheral angiographic features of vasculopathy in idiopathic inflammatory myopathies (IIM) and systemic sclerosis (SSc) in comparison to polyarteritis nodosa (PAN). Angiograms of 47 extremities (24 upper and 23 lower) of 11 patients with IIM (n = 5) and SSc (n = 6), and 12 patients with PAN who presented with critical limb ischemia were retrospectively analyzed with regards to the presence of stenosis, occlusion, aneurysms and delayed distal flow, and degree of neovascularization. Diffuse narrowing was more frequent (66.1 vs. 38.0%, *p* = 0.001), whereas multifocal stenosis (6.5% vs. 26.8%, *p* = 0.002), abrupt occlusion (11.3% vs. 29.6%, *p* = 0.010) and aneurysm formation (1.6% vs. 11.3%, *p* = 0.037) were less frequent in IIM/SSc than PAN. In distal arteries, tapered occlusion (95.5% vs. 76.0%, *p* = ns) and delayed flow (77.3% vs. 48.0%, *p* = 0.039) were more common in IIM/SSc than PAN. After 1 year, auto- or surgical amputation tended to be more frequent in IIM/SSc than PAN (36.4% vs. 16.7%, *p* = ns). In conclusion, diffuse narrowing, tapered occlusion and delayed distal flow on conventional angiograms tend to be more frequent in IIM/SSc than PAN. Further studies are needed to verify these findings in a larger prospective cohort.

## Introduction

Vasculopathies are a heterogeneous group of morphologically and pathogenetically distinct vascular diseases, and can include both non-inflammatory and inflammatory vasculopathies^1^. A key pathologic finding in a patient with a vasculopathy is luminal stenosis and/or occlusion with resulting tissue ischemia, which clinically manifests as pain and ischemic damage of affected organs^2,3^.

In inflammatory vasculopathy (vasculitis) such as polyarteritis nodosa (PAN), the vascular walls are destroyed by infiltrating pro-inflammatory immune cells^4^. Interestingly, patients with connective tissue disease (CTD), such as idiopathic inflammatory myopathies (IIM) and systemic sclerosis (SSc), can experience a non-thrombotic proliferative vasculopathy (NTPV), a distinctive disease entity characterized by vascular wall proliferation without overt evidence of inflammation and thrombosis^5,6^. NTPV in IIM including dermatomyositis (DM) and polymyositis (PM) and SSc commonly affects the pulmonary and extremity arteries resulting in pulmonary arterial hypertension (PAH), secondary Raynaud’s phenomenon, and digital ulcer and gangrene^7,8^.

The nature of the insult to vascular endothelial and mural cells might induce different cascades of inflammatory and metabolic responses. Destruction and subsequent remodeling of affected vessels can be observed as different histologic and angiographic changes^9^. For example, histologic hallmarks of PAN include necrotizing vasculitis of medium-sized muscle arteries, accompanied by segmental transmural inflammation and destruction, fibrinoid necrosis and disruption of elastic lamina^10^. These vascular changes manifest on angiographic images as irregular constriction, occlusion and multiple aneurysms^11^. In addition, a complex network of inflammatory cytokines and ischemia-induced vascular growth factors such as vascular endothelial growth factor (VEGF) induce new vessel formation that manifests as tortuous, corkscrew-like elongation of arteries^12^. By contrast, non-inflammatory proliferative vasculopathy is mediated by an imbalance between proliferative and anti-proliferative signaling in the cells of the intimal and medial wall layers, leading to progressive obliterating thickening of the vascular walls; inflammatory infiltrates, however, are rare and the internal elastic lamina remains intact^13,14^.

To date, the angiographic features of IIM/SSc-vasculopathy have not been fully elucidated. The current study was designed to assess the angiographic characteristics of vasculopathies associated with IIM/SSc in patients, who presented with critical limb ischemia, in comparison to PAN.

## Methods

### Patients

Patients with IIM/SSc, who underwent conventional angiography of an upper and/or lower extremity for critical limb ischemia between January 2001 and May 2020, were included in this retrospective study. The symptoms of critical limb ischemia included Raynaud’s phenomenon with severe pain or numbness, irreversible digital cyanosis, ulcer or gangrene. IIM was diagnosed according to the 2017 American College of Rheumatology (ACR)/European League Against Rheumatism (EULAR) classification criteria for probable or definite DM or PM^15^, whereas SSc was diagnosed according to the 2013 ACR/EULAR classification criteria^16^; and PAN was diagnosed according to the 1990 ACR criteria^17^. We excluded the patients with IIM/SSc who were clinically or histologically diagnosed with other vasculitis (such as anti-neutrophil cytoplasm antibody associated vasculitis or cryoglobulinemic vasculitis). The study was approved by the Institutional Review Board of Seoul National University Hospital (IRB # 2004-081-1117), which waived the requirement for patient informed consent due to the retrospective design of this study. The study was performed in accordance with the principles of the Declaration of Helsinki and Good Clinical Practice guidelines.

### Data collection

The baseline demographic, clinical and laboratory data at the time of angiographic studies were retrieved from the electronic medical records of Seoul National University Hospital. Data recorded include patient age, gender, symptom duration (time between the first ischemic or non-ischemic symptom, and angiography) and disease duration (time between the diagnosis of IIM/SSc or PAN and angiography), comorbidities (cardiovascular risk factors such as hypertension, diabetes, dyslipidemia and smoking status, and malignancy), treatment, and 1-year outcomes.

### Image acquisition

Conventional angiography was performed with ultrasonography-guided transfemoral approach. A 5-French introducer sheath was placed in the femoral artery. A 5-Fr catheter was advanced to the proximal part of each limb under fluoroscopic guidance. The limb was divided into 3 to 4 segments and digital subtraction angiography was conducted respectively with a non-ionic contrast medium (Visipaque 270; GE Healthcare) injected at a rate of 4 mL/s. The injection duration was 3 to 5 s, and adjusted depending on the distance between the catheter tip and region of interests. The images were obtained at 1 frame/s, and recording time was prolonged in case of slow blood flow.

### Angiographic descriptions of the upper and lower extremities

**Figure 1:**
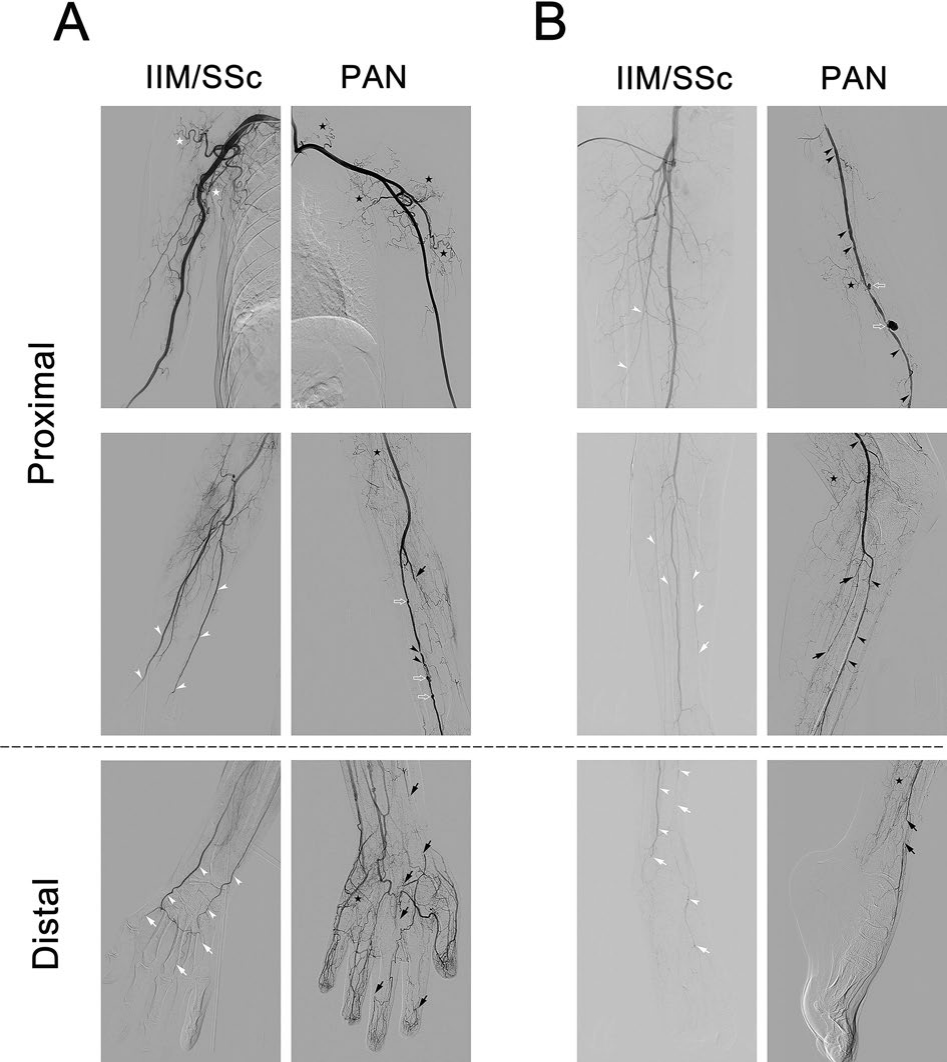
Angiographic features of IIM/SSc-vasculopathy and PAN. Arteries in the (**A**) upper extremities and (**B**) lower extremities of patients with IIM/SSc-vasculopathy and PAN. Diffuse narrowing is indicated by white arrowheads; tapered occlusion by white arrows; multifocal stenosis by black arrowheads; abrupt occlusion by black arrows; aneurysmal changes by empty arrows; grade 2 tortuosity by white stars; and grade 3 tortuosity by black stars. *IIM* idiopathic inflammatory myopathies, *PAN* polyarteritis nodosa, *SSc* systemic sclerosis.

Arteries in the arm were divided into those of the shoulder, elbow and wrist/hand regions, whereas arteries in the leg were divided into those of the thigh, knee, and ankle/foot regions. Peripheral arteries above the wrist or ankle were classified as proximal arteries, whereas those at or below the wrist or ankle were classified as distal arteries. Examples of representative angiograms are shown in Fig. 1.

Each region was evaluated for arterial stenosis (diffuse narrowing, and focal or multifocal stenosis), occlusion (tapered or abrupt), and aneurysmal changes according to the angiographic assessment of central nervous system vasculopathy and allograft vasculopathy^18,19^. The degree of neovascularization in muscle branches was analyzed using a three-point scale, with 1 representing normal to mild (kinking defined as > 60°); 2 representing a moderate degree of tortuosity (30–60°); and 3 representing a severe degree of tortuosity (hypertortuosity; < 30°), using criteria for the tortuosity of the internal carotid arteries^20^. Delayed blood flow of distal arteries was defined by the absence of arterial filling after prolonged recording time (> 1 s) in following arteries: digital and palmar arch arteries in wrist/hands; and pedal plantar arteries in ankle/foot images^21^.

All angiograms were reviewed on a picture archiving and communication system (PACS) workstation (Infinitt, Seoul, Korea) independently by two investigators, a trained rheumatologist (JY) and a radiologist (EAP) who were blinded to clinical information. Discrepancies between the two investigators were resolved by consensus.

### Statistical analysis

Continuous variables were reported as mean and standard deviation (SD) or as median and interquartile range (IQR), as appropriate. Categorical variables were reported in absolute numbers and percentages. Continuous variables were compared using Student’s t-tests or Mann–Whitney tests, whereas categorical variables were compared using chi-square tests or Fisher’s exact tests, as appropriate. *P*-values ≤ 0.05 were considered statistically significant. All statistical analyses were performed with SPSS (IBM SPSS version 26.0, IBM Corp., USA).

### Ethics approval and consent to participate

The study was approved by the Institutional Review Board of Seoul National University Hospital (IRB # 2004-081-1117). The study was performed in accordance with the principles of the Declaration of Helsinki.

### Consent for publication

This study has previously been published as an abstract (POS1386) in EULAR 2021 conference.

## Results

### Baseline characteristics of patients

This study included 11 patients with IIM/SSc, consisting of four with DM, one with PM, and six with SSc, and 12 patients with PAN. Baseline demographic and clinical characteristics of patients at angiographic studies are presented in Table 1. The mean age was 58.0 ± 13.0 years in the IIM/SSc group, and 52.4 ± 17.8 years in the PAN group (*p* = ns). 90.9% of the IIM/SSc group and 50.0% of the PAN group (*p* = ns) were women. The median disease duration was 5.7 (2.0–10.0) years in the IIM/SSc group and 3.7 (0.8–10.0) years in the PAN group (*p* = ns). IIM/SSc patients (5.7 (1.0–9.4) years) had a longer symptom duration of digital ischemia than PAN patients (0.4 (0.1–1.7) years, *p* = 0.002). Digital pain or numbness were reported in 2 (18.2%) of the IIM/SSc group, and 6 (50.0%) of the PAN group (*p* = ns). There were 5 (45.5%) patients with digital gangrene in the IIM/SSc group, as compared to none in the PAN group (*p* = 0.014). Cardiovascular risk factors, such as hypertension, diabetes mellitus, and dyslipidemia, did not differ in these two groups. The average daily glucocorticoid doses at baseline were similar in the two groups (0.3 (0.0–0.3) mg/kg/day in the IIM/SSc group vs. 0.2 (0.0–1.6) mg/kg/day in the PAN group, *p* = ns). Anti-nuclear antibodies of titer ≥ 1:80 were observed in seven (63.6%) patients with IIM/SSc but in none with PAN.

**Table 1:** Baseline demographic and laboratory characteristics of patients.

Characteristics	IIM/SSc (n = 11)	PAN (n = 12)	*p*-value
Age, years	58.0 ± 13.0	52.4 ± 17.8	0.404
Women, n (%)	10 (90.9)	6 (50.0)	0.069
Disease duration, years	5.7 (2.0–10.0)	3.7 (0.8–10.0)	0.487
Smoking history, n (%)	0 (0.0)	3 (25.0)	0.217
Body mass index, kg/m^2^	20.6 ± 2.9	24.0 ± 2.9	**0.011**
Systolic blood pressure, mmHg	132.7 ± 18.4	135.2 ± 23.5	0.786
Diastolic blood pressure, mmHg	86.4 ± 11.2	83.4 ± 17.9	0.644
Symptom duration of digital ischemia, years	5.7 (1.0–9.4)	0.4 (0.1–1.7)	**0.002**
**Manifestations of digital ischemia, n (%)**			
RP with digital pain or numbness	2 (18.2)	6 (50.0)	0.193
Digital cyanosis	3 (27.3)	4 (33.3)	1.000
Digital ulcer	1 (9.1)	2 (16.7)	1.000
Digital gangrene	5 (45.5)	0 (0.0)	**0.014**
**Comorbidity, n (%)**			
Hypertension	4 (36.4)	4 (33.3)	1.000
Diabetes mellitus	1 (9.1)	2 (16.7)	1.000
Dyslipidemia	1 (9.1)	3 (25.0)	0.217
Hepatitis B/C	0 (0.0)	3 (25.0)	0.590
Interstitial lung disease	4 (36.4)	0 (0.0)	**0.037**
Pulmonary hypertension	4 (36.4)	0 (0.0)	**0.037**
Malignancy^a^	1 (9.1)	2 (16.7)	1.000
**Laboratory findings**			
ESR mm/hr	39.3 ± 20.7	41.8 ± 38.2	0.850
CRP, mg/dL	0.46 (0.12–1.04)	0.87 (0.14–2.65)	0.347
Glucose, mg/dL	88.0 (83.0–96.0)	110.0 (88.3–137.3)	**0.032**
Total cholesterol, mg/dL	171.0 (151.0–257.0)	149.0 (119.0–177.0)	0.171
Triglyceride, mg/dL, (n = 7/7)	129.1 ± 76.8	110.7 ± 76.7	0.661
LDL cholesterol, mg/dL (n = 7/8)	117.4 ± 39.3	105.8 ± 39.8	0.580
HDL cholesterol, mg/dL (n = 7/7)	56.0 ± 23.5	55.0 ± 25.8	0.941
D-dimer, ug/mL, (n = 6/11)	1.2 (0.9–2.2)	0.5 (0.3–0.9)	**0.020**
**Autoantibodies positivity, n (%)**			
Anti-nuclear antibody (≥ 1:80)	7 (63.6)	0 (0.0)	**0.001**
Anti-neutrophil cytoplasmic antibody (≥ 1:40)	0/8 (25.0)	0/9 (0.0)	0.206
Anti-Jo-1 antibody	1/4 (25.0)	–	–
Anti-centromere antibody	5/10 (50.0)	0/12 (0.0)	**0.010**
Anti-Scl-70 antibody	3/11 (27.3)	–	–
Anti-RNP antibody	3/6 (50.0)	0/2 (0.0)	0.464
Rheumatoid factor	3/9 (33.3)	0/12 (0.0)	0.063
Anti-cardiolipin antibody IgG/M	0/6 (0.0)	1/10 (1.0)	1.000
Anti-B2GPI antibody IgG/M	0/6 (0.0)	0/8 (0.0)	–
Lupus anticoagulant	0/7 (0.0)	3/11 (27.2)	0.245
Cryoglobulin	1/6 (16.7)	1/9 (11.1)	1.000
**Immunosuppressant drugs use, ever, n (%)**			
Azathioprine	0 (0.0)	7 (58.3)	**0.005**
Cyclosporin	5 (45.5)	0 (0.0)	**0.014**
Mycophenolate mofetil	3 (27.3)	1 (8.3)	0.317
Methotrexate	1 (9.1)	1 (8.3)	1.000
Tacrolimus	1 (9.1)	0 (0.0)	0.478
Cyclophosphamide	1 (9.1)	5 (41.7)	0.155
Rituximab	1 (9.1)	0 (0.0)	0.478
Glucocorticoid use, current, n (%)	6 (54.5)	9 (75.0)	0.400
Cum prednisone-equivalent dose, mg^b^	478.8 (0.0–12,435.0)	158.0 (6.3–828.1)	0.566
Cum prednisone-equivalent dose, mg/kg^b^	9.0 (0.0–231.8)	2.5 (0.1–13.1)	0.347
Average dose of prednisone-equivalent, mg/kg/day^b^	0.3 (0.0–0.3)	0.2 (0.0–1.6)	0.379

Data are expressed as mean ± SD or median (IQR) for continuous variables and number (%) of patients for categorical variables.

Significant values are in bold.

*B2GPI* beta2 glycoprotein I, *CRP* C-reactive protein, *Cum* cumulative, *ESR* erythrocyte sediment rate, *HDL* high-density lipoprotein, *IIM* idiopathic inflammatory myopathies, *LDL* low-density lipoprotein, *PAN* polyarteritis nodosa, *RNP* ribonucleoprotein, *RP* Raynaud’s phenomenon, *SSc* systemic sclerosis.

^a^One colorectal cancer in IIM/SSc group and two hepatocellular carcinoma in PAN group.

^b^The prednisone-equivalent dose includes oral and intravenous glucocorticoids.

### Angiographic characteristics of vasculopathy associated with IIM/SSc

**Figure 2:**
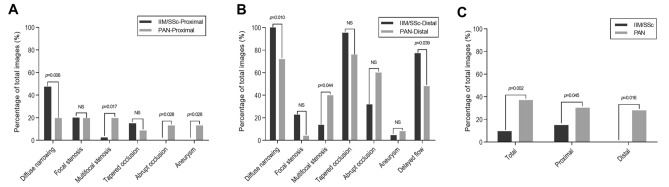
Angiographic parameters in the extremities of patients with IIM/SSc-vasculopathy and PAN. Angiographic features in arteries of the (**A**) proximal and (**B**) distal extremities of patients with IIM/SSc-vasculopathy and PAN. (**C**) Frequencies of neovascularization (hypertortuosity, grade 3) between the IIM/SSc-vasculopathy and PAN. *IIM* idiopathic inflammatory myopathies, *PAN* polyarteritis nodosa, *SSc* systemic sclerosis. Y-axis is the percentage (%) of total images.

Sixty-two angiographic images, 40 of the proximal portion and 22 of the distal portion, were analyzed in the patients with IIM/SSc, whereas 71 images, 46 of the proximal portion and 25 of the distal portion, were analyzed in the patients with PAN (Table 2 and Supplementary Table S1). Diffuse narrowing was significantly more frequent (66.1% vs. 38.0%, *p* = 0.001), whereas multifocal stenosis was significantly less common (6.5% vs. 26.8%, *p* = 0.002) in the IIM/SSc-vasculopathy than in the PAN. The proximal arteries showed similar findings; diffuse narrowing (47.5% vs. 19.6%, *p* = 0.006) was more common and multifocal stenosis (2.5% vs. 19.6%, *p* = 0.017) was less common in the IIM/SSc than in the PAN group (Fig. 2A). Notably, all patients in the IIM/SSc group, compared with 72.0% in the PAN group, had diffuse narrowing in distal arteries (*p* = 0.010) (Fig. 2B).

**Table 2:** Comparison of angiographic parameters between IIM/SSc-vasculopathy and PAN.

	IIM/SSc-vasculopathy (upper 14, lower 8)	PAN (upper 10, lower 15)	*p*-value
Total number of images	62	71	
Shoulder/elbow/wrist and hand	11/14/14	8/10/10	
Femoral/knee/ankle and foot	7/8/8	13/15/15	
**Stenosis**			
Diffuse narrowing	41/62 (66.1%)	27/71 (38.0%)	**0.001**
Focal stenosis	13/62 (21.0%)	10/71 (14.1%)	0.295
Multifocal stenosis	4/62 (6.5%)	19/71 (26.8%)	**0.002**
**Occlusion**			
Tapered occlusion	27/62 (43.5%)	23/71 (32.4%)	0.185
Abrupt occlusion	7/62 (11.3%)	21/71 (29.6%)	**0.010**
Aneurysm	1/62 (1.6%)	8/71 (11.3%)	**0.037**
**Neovascularization in muscular branch**			
Tortuosity^a^			**0.002**
Grade 1	45/62 (72.6%)	30/68 (39.1%)	
Grade 2	11/62 (17.7%)	14/68 (23.9%)	
Grade 3	6/62 (9.7%)	24/68 (37.0%)	

*IIM* idiopathic inflammatory myopathies, *PAN* polyarteritis nodosa, *SSc* systemic sclerosis.

Significant values are in bold.

^a^Tortuosity grade 1, normal; grade 2, mild to moderate; grade 3, severe (hypertortuosity). Data are expressed as number (%) of images.

Abrupt occlusion (11.3% vs. 29.6%, *p* = 0.010) and aneurysm formation (1.6% vs. 11.3%, *p* = 0.037) were significantly less frequent in the IIM/SSc-vasculopathy than in the PAN group. Interestingly, both abrupt occlusion and aneurysm formation did not involve proximal arteries in patients with IIM/SSc-vasculopathy (Fig. 2A). In addition, a higher degree of tortuosity (grade 3) was significantly less frequent in the IIM/SSc-vasculopathy than in the PAN group (9.7% vs. 37.0%, *p* = 0.002) (Fig. 2C). In most IIM/SSc vasculopathy showed tapered occlusion in distal arteries, while PAN showed lower frequency, albeit not significantly (95.5% vs. 76.0%, *p* = ns) (Fig. 2B). Strikingly, poor overall distal blood flow was more frequent in patients with IIM/SSc-vasculopathy than PAN (77.3% vs. 48.0%, *p* = 0.039) (Fig. 2B). In the subgroup analysis of the IIM/SSc-vasculopathy, angiographic characteristics showed no significant difference between patients with IIM and those with SSc (Table 3).

**Table 3:** Comparison of angiographic parameters in subgroups of IIM/SSc-vasculopathy.

	IIM-vasculopathy (upper 8, lower 3)	SSc-vasculopathy (upper 6, lower 5)	*p*-value
Total number of images	31	31	
Shoulder/elbow/wrist and hand	6/8/8	5/6/6	
Femoral/knee/ankle and foot	3/3/3	4/5/5	
**Stenosis**			
Diffuse narrowing	21/31 (67.7%)	20/31 (64.5%)	0.788
Focal stenosis	6/31 (19.4%)	7/31 (22.6%)	0.755
Multifocal stenosis	2/31 (6.5%)	2/31 (6.5%)	1.000
**Occlusion**			
Tapered occlusion	13/31 (41.9%)	14/31 (45.2%)	0.798
Abrupt occlusion	5/31 (16.1%)	2/31 (6.5%)	0.425
Aneurysm	0/31 (0.0%)	1/31 (3.2%)	1.000
**Neovascularization in muscular branch**			
Tortuosity^a^			0.407
Grade 1	20/31 (64.5%)	25/31 (80.6%)	
Grade 2	7/31 (22.6%)	4/31 (12.9%)	
Grade 3	4/31 (12.9%)	2/31 (6.5%)	

Data are expressed as number (%) of images.

*IIM* idiopathic inflammatory myopathies, *SSc* systemic sclerosis.

^a^Tortuosity grade 1, normal; grade 2, mild to moderate; grade 3, severe (hypertortuosity).

### Treatment of digital ischemia and clinical outcomes

Treatment and 1-year outcome of limb ischemia are summarized in Table 4. Of the patients with IIM/SSc, eight (72.7%) and nine (81.8%) were treated with calcium channel blockers and prostanoids, respectively. The frequency of treatment with antithrombotic drugs did not differ significantly in the IIM/SSc and PAN groups (*p* = ns). After 1 year, three (27.3%) patients in the IIM/SSc group and seven (58.3%) in the PAN group showed improvements in digital ischemia. Auto- or surgical amputation tended to be more frequent in the IIM/SSc than in the PAN groups (36.4% vs. 16.7%, *p* = ns).

**Table 4:** Treatment and 1-year outcome of limb ischemia.

	IIM/SSc-vasculopathy (n = 11)	PAN (n = 12)	*p*-value
**Vasoactive drugs**			
Calcium channel blocker	8 (72.7)	3 (25.0)	**0.022**
PDE5 inhibitor	5 (45.5)	0 (0.0)	**0.014**
Prostanoid	9 (81.8)	5 (41.7)	0.089
Endothelin receptor antagonist	3 (27.3)	0 (0.0)	0.093
**Antithrombotic drugs**			
Aspirin	5 (45.5)	5 (41.7)	1.000
Clopidogrel	1 (9.1)	1 (8.3)	1.000
Cilostazol	4 (36.4)	5 (41.7)	1.000
Warfarin	1 (9.1)	1 (8.3)	1.000
Direct oral anticoagulant	1 (9.1)	0 (0.0)	0.478
**Surgical procedure, n (%)**			
Skin graft	1 (9.1)	0 (0.0)	0.478
Amputation	2 (18.2)	1 (8.3)	0.590
Bypass surgery	0 (0.0)	1 (8.3)	1.000
PTA, n (%)	1. (9.1)	1 (8.3)	1.000
**1-year outcome, n (%)**			
Improved	3 (27.3)	7 (58.3)	0.214
Stationary	4 (36.4)	3 (25.0)	0.667
Amputation (auto- or surgical)	4 (36.4)	2 (16.7)	0.371

Data are expressed as number (%) of patients.

Significant values are in bold.

*IIM* idiopathic inflammatory myopathies, *PAN* polyarteritis nodosa, *PDE* phosphodiesterase, *PTA* percutaneous transluminal angioplasty, *SSc* systemic sclerosis.

## Discussion

To our knowledge, this study is the first to compare the angiographic involvement of arteries in the extremities of IIM/SSc patients with severe limb ischemia and PAN patients. This study found that diffuse narrowing, tapered occlusion and delayed distal blood flow tended to be more common in IIM/SSc patients, whereas multifocal stenosis, abrupt occlusions, aneurysmal changes and tortuous hypervascularization were more common in patients with PAN. In addition, angiographic findings did not differ between IIM and SSc.

The angiographic characteristics of IIM/SSc-vasculopathy have not been fully elucidated. As a secondary Raynaud's phenomenon with endothelial dysfunction was thought to account for digital ischemia and ulcer/gangrene in patients with IIM/SSc, these patients do not routinely undergo peripheral angiography^22^. We and others reported previously that patients with SSc had structural changes involving the digital arteries, as well as the distal ulnar and radial arteries^23–26^. The current study unequivocally showed that also IIM/SSc-vasculopathy involves more proximal arteries as well. The angiographic features of IIM/SSc-vasculopathy included diffuse, segmental narrowing with distal tapering, with multifocal stenosis and abrupt occlusion being less frequent in IIM/SSc than in PAN. This angiographic pattern of vasculopathy in IIM/SSc-vasculopathy are consistent with the abnormal vascularity, smaller arterial lumens, and thickened artery walls observed on color-Doppler ultrasound in patients with secondary Raynaud's phenomenon^27,28^. The diffuse and extensive vasculopathy in proximal and distal arteries might account for the delayed or absent flow in the distal arteries, including the palmar and plantar arches. This may also explain the greater susceptibility of patients with IIM/SSc to severe “watershed” ischemia/damage during Raynaud’s attacks^29,30^. Angiographic findings did not differ between groups of patients with IIM and SSc, suggesting that the mechanisms of vasculopathy are similar in these diseases. Interestingly, PAN was frequently accompanied by a high degree of tortuosity of muscle arteries, suggesting that the production of angiogenic cytokines during active vasculitis drives new vessel formation in synergy with VEGF^12^.

Histologically, IIM/SSc-associated vasculopathy is characterized by luminal stenosis/occlusion with hyperplasia of mural cells^31^, which may explain the long-segmental narrowing without aneurysm formation observed on angiograms of patients with IIM/SSc. By contrast, the vascular walls in patients with PAN were destroyed by infiltrating immune cells, which can be observed on angiograms as abrupt occlusion and aneurysm formation.

Patients with IIM/SSc-vasculopathy tended to have poorer outcomes than those with PAN, as shown by a numerically higher rate of auto- and surgical amputation (36.4% vs. 16.7%, *p* = ns) during 1-year follow-up. It is not clear if IIM/SSc-vasculopathy would respond to more aggressive anti-inflammatory treatment, since there is little histologic evidence of inflammation. Therefore, IIM/SSc-vasculopathy may require a treatment targeting “pauci-inflammatory” vascular remodeling. Studies are needed to address the therapeutic roles of anti-proliferative drugs, such as endothelin receptor antagonist (used in the treatment of PAH) and paclitaxel (used to prevent restenosis of coronary artery after percutaneous coronary intervention)^32–34^. Since different immunosuppressants were used in IIM/SSc and PAN groups, it is difficult to evaluate the effect of immunosuppressive therapies on angiographic changes in two groups.

Patients with systemic lupus erythematosus (SLE) were intentionally excluded in this study since they are more likely to develop both inflammatory and thrombotic (due to antiphospholipid syndrome) vasculopathies^35^. Therefore, evaluation of SLE vasculopathy is needed in further studies.

This study had several limitations, due in part to its retrospective design. First, the relatively small number of patients limits the ability to generalize the current findings to all patients with IIM/SSc-vasculopathy. Especially, the relationship between vasculopathy and myositis-specific autoantibodies including anti-NXP2 and anti-MDA5, which may confer risk of vasculopathy^36^, and underlying diseases such as PAH and malignancy needs to be determined. Second, all the included patients had more severe ischemic symptoms requiring angiographic evaluation, leading to a selection bias. Third, conventional angiography cannot accurately differentiate NTPV from other forms of vascular abnormalities including vasculitis, atherosclerosis or thrombosis. Fourth, follow-up angiograms would help to determine whether the angiographic findings in the present study are reversible or progress with or without treatment. Finally, biopsy of affected arteries are needed to correlate histopathologic findings with angiographic abnormalities. Studies in larger patient cohorts are needed to determine the prevalence of and risk factors for IIM/SSc-vasculopathy, as well as long-term outcomes.

## Conclusions

The present study showed that diffuse narrowing, tapered occlusion and delayed distal blood flow on conventional angiograms tend to be more frequent in patients with IIM/SSc-vasculopathy than with PAN. Larger studies are needed to validate the current findings.

## Supplementary Information

Supplementary Information.

## Data Availability

The data in this article are available from the corresponding authors upon reasonable request.
