# Supplementary material for: Angiographic characteristics of vasculopathy in patients with idiopathic inflammatory myopathies and systemic sclerosis
Source: Sci Rep. 2022 May 31;12:9051. doi: 10.1038/s41598-022-12991-y (PMC9156678; doi:10.1038/s41598-022-12991-y)
Supplement: Supplementary file 1 — Supplementary Information. [file 41598_2022_12991_MOESM1_ESM.pdf]

Supplementary data

Title: Angiographic characteristics of vasculopathy in patients with idiopathic inflammatory myospathies and systemic sclerosis.

Authors: Jina Yeo, Eun-Ah Park, Eun Bong Lee, and Jin Kyun Park*

Table of Contents

Supplementary Table S1. **Page 2**

	IIM/SSc-vasculopathy (upper 14, lower 8)	PAN (upper 10, lower 15)	*p*-value
**Total number of images**	62	71	
**Shoulder/elbow/wrist and hand**	11/14/14	8/10/10	
**Femoral/knee/ankle and foot**	7/8/8	13/15/15	
**Proximal arteries**[a]			
**Stenosis**			
Diffuse narrowing	19/40 (47.5%)	9/46 (19.6%)	**0.006**
Focal stenosis	8/40 (20.0%)	9/46 (19.6%)	0.960
Multifocal stenosis	1/40 (2.5%)	9/46 (19.6%)	**0.017**
**Occlusion**			
Tapered occlusion	6/40 (15.0%)	4/46 (8.7%)	0.504
Abrupt occlusion	0/40 (0.0%)	6/46 (13.0%)	**0.028**
**Aneurysm**	0/40 (0.0%)	6/46 (13.0%)	**0.028**
**Neovascularization** in muscular branch			
**Tortuosity**[b]			**0.045**
Grade 1	25/40 (62.5%)	18/46 (50.0%)	
Grade 2	9/40 (22.5%)	11/46 (19.6%)	
Grade 3	6/40 (15.0%)	17/46 (30.4%)	
**Distal arteries**[a]			
**Stenosis**			
Diffuse narrowing	22/22 (100.0%)	18/25 (72.0%)	**0.010**
Focal stenosis	5/22 (22.7%)	1/25 (4.0%)	0.085
Multifocal stenosis	3/22 (13.6%)	10/25 (40.0%)	**0.044**
**Occlusion**			
Tapered occlusion	21/22 (95.5%)	19/25 (76.0%)	0.102
Abrupt occlusion	7/22 (31.8%)	15/25 (60.0%)	0.053
**Aneurysm**	1/22 (4.5%)	2/25 (8.0%)	1.000
**Delayed blood flow**	17/22 (77.3%)	12/25 (48.0%)	**0.039**
**Neovascularization** in muscular branch			
**Tortuosity**[b]			**0.016**
Grade 1	20/22 (90.9%)	15/25 (60.0%)	
Grade 2	2/22 (9.1%)	3/25 (12.0%)	
Grade 3	0/22 (0.0%)	7/25 (28.0%)	

Supplementary Table S1. Comparison of angiographic parameters according to proximal or distal portion between IIM/SSc-vasculopathy and PAN.

[a]Peripheral arteries above the wrist or ankle were classified as proximal arteries, whereas those at or below the wrist or ankle were classified as distal arteries.

[b]Tortuosity grade 1, normal; grade 2, mild to moderate; grade 3, severe (hypertortuosity).

IIM, idiopathic inflammatory myopathies; PAN, polyarteritis nodosa; SSc, systemic sclerosis
